# Artificial intelligence in the workplace: a living systematic review protocol on worker safety, health, and well-being implications

**DOI:** 10.1186/s13643-025-03000-0

**Published:** 2025-12-30

**Authors:** Arif Jetha, Meghan Crouch, Karina Vold, Susan Elizabeth Peters, Jay Vietas, Abi Sriharan, Emma Irvin

**Affiliations:** 1 Institute for Work & Health, Toronto, ON Canada; 2 Dalla Lana School of Public Health, University of Toronto, Toronto, ON Canada; 3 Institute for the History and Philosophy of Science and Technology, University of Toronto, Toronto, ON Canada; 4 Center for Work, Health and Well-Being, Harvard T.H. Chan School of Public Health, Harvard University, Cambridge, MA USA; 5 National Safety Council, Itasca, Illinois USA; 6 Krembil Centre for Health Management and Leadership, Schulich School of Business, York University, Toronto, ON Canada; 7 Institute of Health Policy, Management and Evaluation, University of Toronto, Toronto, ON Canada

**Keywords:** Artificial intelligence, Systematic reviews, Occupational health, Employment, Working conditions

## Abstract

**Background:**

Advancements in artificial intelligence (AI) are transforming employment and working conditions in ways that shape the safety, health, and well-being of workers. We describe a protocol for a living systematic review (LSR) that will examine the interrelationship between AI systems, employment and working conditions, and worker safety, health, and well-being. Research questions are:

1. What types of AI systems are being used within workplaces and how do their design and adoption impact worker safety, health, and well-being?
2. How do a worker’s employment and working conditions affect the relationship between the adoption of AI systems and worker safety, health, and well-being?
3. How does a worker’s social position (e.g., age, gender, race, disability) shape the interrelationship between AI systems at work, employment and working conditions, and their safety, health, and well-being?

**Methods:**

A comprehensive search of primary qualitative and quantitative research will be conducted. MEDLINE, Embase (OVID), PsycINFO (OVID), and Web of Science will be searched every six to twelve months using database-specific terms and keywords. Title/abstract and full-text screening will be completed independently by two reviewers. Relevant articles will be quality appraised using a mixed method assessment tool adapted for studies of AI. Medium and high-quality studies will be synthesized using a best evidence synthesis approach. To ensure relevancy, applied workplace and AI stakeholders will provide feedback at all stages of the LSR process through dissemination excluding quality appraisal. Annually, we will evaluate the appropriateness of the review process (e.g., frequency of searches, requirement to refine research questions, utility of continuing LSR). Any amendments to protocols will be documented.

**Discussion:**

This LSR will provide timely and evolving evidence on the implications of AI in the workplace that will be disseminated through a publicly available living review dashboard. We will capture the emerging impact AI has on workers. Findings can be used to develop strategies to minimize AI’s potential workplace harms while amplifying its potential benefits, address emerging worker inequities, and inform ongoing discussions regarding responsible and safe AI adoption.

**Systematic review registration:**

PROSPERO CRD42024625501.

**Supplementary Information:**

The online version contains supplementary material available at 10.1186/s13643-025-03000-0.

## Background

Artificial intelligence (AI) is transforming the world of work and has the potential to affect employment and working conditions that are known to directly and indirectly impact worker safety, health, and well-being [1–5]. AI is a series of evolving general-purpose technologies that leverage complex algorithms and big data to perform specific functions traditionally requiring human intelligence. Within the workplace, these functions can include developing predictions, detecting patterns, and automating or augmenting diverse tasks [1, 2, 6]. While much attention has focused on how AI systems will innovate organizational practices across different industries, less has focused on how AI could positively or negatively impact workers including their safety, health, and well-being [5]. There is an urgent need for evidence on AI’s impact on workers to prioritize responsible AI design and implementation and safeguards for workers in a working world where AI is increasingly prominent [5, 7]. In this protocol, we describe a living systematic review (LSR) to conduct an ongoing examination and synthesis of research on the impact of AI on the safety, health, and well-being of workers.

There is an established body of research which highlights the role of employment and working conditions in shaping worker health [8, 9]. Employment conditions include objectively measured dimensions such as contract permanence, wage and non-wage benefits, work hours, and union representation [10–13]. Working conditions include subjective dimensions such as a worker’s perceptions of their job security, job demands and control, and workplace social support [10–13]. Studies consistently show that both employment and working condition factors can be directly or indirectly tied to a worker’s physical and mental health, prevalence of workplace injuries, and perceptions regarding their work (e.g., job stress, job satisfaction) [14–17]. In many cases, employment and working conditions are patterned according to workers’ social position (e.g., age, gender, race, immigration status, disability) with certain labor market subgroups (e.g., women, racialized persons, immigrants, persons living with disabilities) more likely to experience hazardous, precarious, and less supportive conditions at work that can adversely impact safety, health, and well-being [18, 19].

Historically, the introduction of new technologies has largely created economic opportunities and enhanced productivity [20–22]. New technologies have also played a role in changing employment and working conditions that may be both beneficial and harmful to the health of the working population [8, 9, 23]. Unlike previous technologies, AI systems are being implemented within workplaces (often in conjunction with other technologies) at a faster rate and greater scale and therefore have the potential to amplify challenges and opportunities for worker health in both expected and unanticipated ways [24–26]. AI is an umbrella term broadly referring to the use of computing machines to solve problems traditionally requiring human intelligence, including detecting patterns, making predictions and decisions, or optimizing processes [3, 27–29]. Central to all AI are algorithms (sequences of mathematical operations) trained on diverse forms of data. Under the AI umbrella include machine and deep learning, and large language models (LLMs) which utilize algorithms to imitate human capabilities [3, 28, 30–32]. In its current form, AI is not expected to fully automate occupations. Instead, job tasks are more likely to be redistributed, and workers’ efforts augmented with AI performing specific tasks [1, 33].

To date, most research on workplace adoption of AI has focused on organizational outcomes, such as productivity, performance, and innovation [34]. There is a minimal amount of research on the implications of AI systems for worker health [5, 7]. Studies that do exist highlight several potential positive and negative implications for workers. For instance, AI systems may be used by workplaces to automate strenuous or mundane job tasks (e.g., data entry, email responses, medical notetaking), free up time for workers to perform high value activities (e.g., client facing engagement, coworker collaboration), and contribute to improved job performance [35–37]. The use of AI tools by organizations for management purposes (e.g., to monitor workers) can provide real-time feedback that improves productivity and may be used to identify hazards at work but may also increase fears of surveillance and loss of privacy [34, 38, 39]. Further, the automation of job tasks can raise workers’ concerns over job security or lost wages [37, 40]. Working alongside AI can contribute to work intensification, performance pressures, and stress or burnout for workers required to keep up with the technology [36, 41–43]. AI tools can also adversely affect perceptions of job control and agency [44, 45]. Within this research it is unclear the extent to which AI systems at work impact worker safety, health, and well-being outcomes [5]. It is also unclear whether the relationship between the use of AI systems and worker health differs according to workers’ social positions, employment and working conditions [4], and AI design and implementation considerations (e.g., transparency in how tools were generated, AI reliability [46–48]).

According to a recent 2024 global survey of organizational representatives across a variety of industries and firm sizes, 72% of organizations have adopted AI while over two-thirds plan to increase their investments in AI over the next 3 years [49]. AI’s capacity to learn, adapt, and generate outputs with increasing independence and accuracy means that AI has potential applications and benefits across a broad range of industries and occupations [3, 28, 30]. As AI advances in its ability to perform more complex job tasks independently, it is expected to substantially change the nature of work, occupations, job roles, and skill requirements as well as the health of workers [3, 5–7]. The rapid speed of AI innovation and adoption requires a research approach that can be used to track the ongoing impact of the technology on employment and working conditions and their influence on workers’ health.

Recognizing the impact of AI on work, there is growing scholarly and policy attention towards safe and responsible AI design which focuses on developing, deploying, and using AI systems in ways that are ethical, transparent, accountable, and have societal benefits [7, 43, 50]. The goal is to create AI systems that are aligned with human values, respect fundamental rights, and promote the well-being of individuals and society. Given the societal implications of AI including its impact on work and health, it is not surprising that a growing number of governmental and international organizations are taking steps to develop policies that advance responsible and safe AI [7]. To inform policy discussions and deliberations, there is a critical need to synthesize up to date evidence on the impact of workplace-based AI systems on the safety, health, and well-being of the working population.

Within the context of AI advancement and workplace adoption, the aim of this LSR protocol is to conduct an ongoing identification and synthesis of research regarding the impact of workplace-based AI on worker safety, health, and well-being. Using an LSR design, we will answer the following research questions:

1. What types of AI systems are being used within workplaces and how do their design and adoption impact worker safety, health, and well-being?
2. How do a worker’s employment and working conditions affect the relationship between the adoption of AI systems and worker safety, health, and well-being?
3. How does a worker’s social position shape the interrelationship between AI systems at work, employment and working conditions, and their safety, health, and well-being?

Given the early stages of research on the work and health implications of AI, our research questions are purposely broad. As the LSR progresses, and depending on the body of literature we identify, we will refine the precision of our research questions.

## Methods

We will conduct a LSR to examine current and emerging evidence on AI systems at work [51]. LSRs draw on standard systematic review methodology that involves the rigorous surveillance, appraisal, and synthesis of literature to provide timely evidence [51, 52]. LSRs are particularly useful to monitor ongoing evidence in an emerging research area where the number of high-quality studies is low but is expected to increase over time [51, 53]. As an example, LSRs gained prominence during the COVID-19 pandemic as a method to synthesize evidence that kept pace with the growth of new research [52, 54]. Given ongoing AI advancement and adoption within workplaces, LSRs are well suited to study the impact of the technology on worker safety, health, and well-being and capture the emergent body of research on the topic over time. Identifying and examining qualitative and quantitative research will enable us to capture multiple perspectives as well as breadth and depth regarding the impact of AI on worker experiences.

### Theoretical foundation

Our LSR draws on a conceptual framework for understanding work as a critical determinant of health [9]. This framework situates work within a broader socioeconomic and political context recognizing that these structural factors affect employment conditions including globalization, cultural and societal values, and technological advancement [8]. The framework identifies workers’ social positions including socioeconomic status, age, ethnicity and race, gender and sexual identity as factors which may impact their exposure to certain employment and physical and psychosocial working conditions. Both employment and working conditions have the potential to be shaped by AI adoption and use and may impact worker safety, health, and well-being contributing to health inequities in the working population. We will utilize this conceptual framework in the design and implementation of our LSR and in the interpretation of findings.

### LSR approach

The development of this LSR protocol was informed by a multidisciplinary research team and engagement with stakeholders that included representatives from government/governmental agencies, civil society organizations, AI organizations, unions, and employers. Specifically, an agenda setting meeting with stakeholders was held in the Fall of 2022 which underscored the importance of an ongoing review of published literature on the health impacts of AI at work and informed the development of our research questions and the methodological approach taken in this review [5]. Applied stakeholder engagement throughout the span of the living review will inform the synthesis and interpretation of findings and provide input on dissemination methods and updating the search strategy. This integrated knowledge transfer and exchange approach makes research evidence available and accessible to inform decision making [55].

The LSR will be conducted over a ten-year period to capture advancements in AI systems and their application within workplaces. Every year, we will review the quality of emerging evidence and the strength of its relationship with the outcomes of interest. Based on the number and consistency of findings and through a consensus-based approach, our team may decide to conduct less frequent searches of the literature or end the LSR altogether. Stakeholder engagement meetings will be held annually to obtain feedback on emerging findings. Engagement meetings will also be used to help us determine whether objectives have been met and the utility of continuing the living review.

In the following sections we will describe the LSR approach in greater detail. The protocol has been registered to PROSPERO (CRD42024625501) and aligns directly with the Preferred Reporting Items for Systematic Reviews and Meta-Analyses Protocols (PRISMA-P) (Additional file 1). Specific LSR protocol elements are reported according to PRISMA-LSR extension guidelines to fully describe the living mode parameters [56].

### Search strategy

The search strategy will follow a Population (P), Exposure (E), Comparator (C), and Outcome (O) (PECO) framework that was directly informed by stakeholder feedback. We will search for primary quantitative (e.g., experimental or observational studies), qualitative (e.g., ethnographic, grounded theory studies), or mixed methods (e.g., explanatory sequential design studies) research which focuses on any group of workers in industrialized middle- or high-income countries including those who are members of the Organisation of Economic Cooperation and Development (OECD) and China, India, and Brazil, where there are high levels of economic activity and growing AI adoption (P)[57, 58]. Our LSR will focus on any AI system that has been implemented within the workplace (E). Physical, remote, or hybrid work environments and specific types of AI systems that use machine learning, neural networks, or LLMs will be included. Our search will include studies with any comparison group (e.g., pre-post, randomized controlled trials) (C) and that measure any worker safety (e.g., work-related physical or mental health injuries, occupational disease), health status (i.e., physical [e.g., acute or chronic conditions] or mental health [e.g., depression, anxiety, burnout]), or well-being (e.g., life satisfaction, flourishing, social support, sense of belonging) outcomes (O). In addition, articles should be published $\ge$ 2019 to reflect the recent prominence of AI-related applications within workplaces over the past 6 years. We will consider studies written in any language.

Our PECO parameters will be translated into database-specific search terms and keywords to search for articles in MEDLINE, Embase (OVID), PsycINFO (OVID), and Web of Science which has been previously identified by our research team as effective in capturing multidisciplinary evidence (see Additional file 2 for example). The set of search terms were developed in collaboration with a library services specialist in consultation with the review team and informed by search strategies from previous reviews on similar topics [59]. The search strategy was extensively reviewed by a second librarian services specialist with expertise in evidence synthesis in the field of occupational health and safety.

Literature searches will be conducted every six to twelve months. We will update the search strategy over the ten-year period by leveraging applied stakeholder expertise and the literature to identify new AI systems and applications and relevant search terms or keywords that can be incorporated into subsequent search protocols. Duplicates will be removed prior to article screening using EndNote. DistillerSR systematic review software will be used to manage all other stages of the review including screening records, quality appraisal, and data extraction.

### Article screening

We will conduct title/abstract and full-text screening that will align with the PECO parameters described in the above paragraphs. Studies will be excluded if the AI application is for hiring (i.e., to support candidate selection or interview process) and may not shape at-work experiences. Studies that do not explicitly describe involvement of an AI system when utilized with other advanced technologies (e.g., industrial robotics, Internet of Things) or do not explicitly report outcome effects for worker safety, health, and/or well-being will be excluded. Also, articles will be excluded if they are secondary research (i.e., reviews), commentaries, editorials, or conference abstracts.

Titles and abstracts of references identified in the search will be assigned to research team members and screened independently by at least two reviewers for relevancy. Any disagreements will be resolved by a third reviewer. Two reviewers will then independently screen the full articles of those studies that meet our relevance criteria. Team members will not review studies they consulted on, authored, or co-authored. Regular team meetings will be organized to discuss discrepancies and resolve disagreements using a consensus-based approach. Reference lists of included articles will be reviewed to identify any potentially relevant articles that were not captured in our search.

Of note, both stages of the screening process will be pilot tested to ensure all team members are consistently applying inclusion and exclusion criteria. Once all reviewers have completed the pilot screening, responses will be compared, and inconsistencies will be reviewed and discussed among the team. Any amendments to screening procedures will be made and updated in the protocols prior to the full screening process.

### Quality appraisal

All relevant articles will be appraised using a quality assessment tool developed for reviews including quantitative and qualitative studies. Currently no existing quality appraisal tools have been developed to appraise diverse studies focusing on worker health impacts of AI. Accordingly, the Mixed Methods Appraisal Tool (MMAT)[60] was selected to assess quality across the sources of evidence we identify in our study. The tool consists of a series of domain specific methodological criteria to assess the quality of either qualitative, quantitative, or mixed methods studies. Qualitative criteria include relevancy of data sources and analysis methods to address research questions and consideration for study context and researcher impact on interpretation of findings. Quantitative criteria are specific to randomized controlled, non-randomized, and descriptive research designs respectively. Mixed methods appraisal criteria include the relevancy of a mixed methods approach and integration of quantitative and qualitative data to address research questions as well as the qualitative and quantitative criteria. Additional questions will be added to the quality assessment tool to examine the extent to which the AI systems in eligible research studies were described including the rationale for AI use.

Each relevant article will be appraised by two independent reviewers using the adapted tool. Through a consensus discussion, for our purposes, we will generate a final weighted sum score of the quality criteria in the MMAT and convert it to a percentage score. Using the percentage scores, studies will be categorized as high (≥ 85%), medium (50%–84%), or low quality (< 50%). To reach consensus on final appraisal scores and rankings, the research team will meet to discuss. Only studies that are appraised as high and medium quality will be included in the evidence synthesis phase.

### Data extraction

Data will be extracted to address core research questions. Data extraction tables will be used to summarize the evidence on the three main research objectives. They will describe the AI system, its impact on worker safety, health, and well-being outcomes, and difference according to employment and working conditions, and a worker’s social position. Descriptive study details including theoretical lens or theories used in the studies and the workplace context (e.g., industry and occupation) will also be documented in summary tables. Key messages and applied recommendations will be generated using the best evidence synthesis approach which has been previously utilized by our team for systematic reviews in the field of occupational health where there is variability in study design, populations, and worker outcomes [61]. The level of evidence needed to generate key messages is based on the methodological quality of studies (i.e., high, medium, or low), quantity of studies (i.e., number of studies with similar sample characteristics and outcome measures), and the consistency of study findings (i.e., number of studies which produce similar findings). Key messages will be developed according to the level of the evidence (i.e., strong, moderate, limited, mixed or insufficient). Strong levels of evidence can be used to generate messages including practice recommendations whereas medium levels of evidence can inform practice considerations. Through peer-reviewed manuscripts, we will publish updated findings from the LSR every second year.

Our proposed research questions guiding the LSR, were intentionally broad in scope to reflect the emergent nature of the literature on this topic. Over time, as the LSR proceeds, the research questions may be refined depending on the state of science and we may be able to begin to categorize and further define the AI workplace systems in use. Additionally, over time, we may also make the decision (based on the level and strength of evidence) to focus on specific industries or occupations where specific AI systems are prominent. All refinements to the evidence synthesis approach will be made by members of the authorship team based on consensus. Any protocol amendments will be updated on PROSPERO and changes will be captured in subsequent publications. If we observe consistency in AI applications and outcomes reported in studies, we may choose to conduct a meta-analysis. We will continually monitor AI systems to which workers are exposed and health outcomes reported in eligible articles. If we reach the threshold of six studies, we will calculate pooled effect estimates.

### Knowledge mobilization strategy

The impact of AI adoption on worker safety, health, and well-being has relevance to a wide variety of stakeholders including workers, employers, unions, policymakers, and AI developers. To facilitate knowledge dissemination and uptake of the emergent evidence, key messages, and recommendations that are useable to inform policy and practice to protect worker safety and health amidst growing AI use, an integrated knowledge transfer and exchange approach will be adopted by our study team [55]. Stakeholders representing diverse perspectives will be involved in the interpretation of findings and generation of key messages to ensure practical implications for different knowledge users are effectively communicated in a variety of products that can be used to address potential health impacts associated with AI.

The development and maintenance of a living review dashboard on a publicly available website maintained by the lead author’s institution will be used to share synthesized evidence and key messages from the first and updated LSR versions, and any new relevant studies as they become available after each update. Depending on the strength of the evidence, other expected knowledge products will include plain language summaries, policy briefs, and technical guidance documents for developers and organizations to design and implement safe and responsible AI for workplace use that emphasizes worker well-being. By including diverse stakeholders with a variety of perspectives from different industries and sectors throughout the process, the aim is to produce relevant and timely knowledge to inform decision making [53].

## Discussion

An AI revolution has the potential to drastically impact the nature and availability of work within a number of contexts [3, 28, 30]. Our research seeks to understand the evolving impact AI may have on the health of the working population. In this manuscript, we described a protocol for a LSR that will build ongoing insights regarding how AI systems impact worker safety, health, and well-being that will take place over a ten-year period. As research on this topic is still in its infancy, our LSR process will provide timely and foundational knowledge regarding the implications of AI in the workplace that keeps pace with AI innovation and the emerging evidence. Given the rapid innovation in AI systems and growing calls to advance responsible AI design and safeguard workers, studying AI’s impact on workers is well suited for the LSR method.

Past research has indicated that the adoption of new technologies within the workplace has created both challenges and opportunities for the health of the workers [8, 23]. AI represents a rapidly advancing technology with an increasing number of applications within the workplace [5, 7]. A LSR is essential to generate an up-to-date evidence base that keeps pace with the adoption and use of different AI systems and applications within the workplace. The ongoing and iterative nature of this LSR will capture the risks and benefits of AI systems to a range of worker health and well-being outcomes while also highlighting where evidence and the certainty of this evidence remain limited.

There is a large body of research highlighting the role of employment and working conditions in shaping the safety, health, and well-being of workers [8, 9, 62, 63]. Our study will elaborate on how AI can impact employment and working conditions in ways that also affect worker safety, health, and well-being. We draw from a contemporary conceptual model in the fields of occupational and public health to interpret findings and elucidate the relationship between AI systems and worker health [9]. Through a focus on employment and working conditions, we will generate evidence that identifies the complexities of AI adoption at work including how the technology can impact employment arrangements and the work environment to which workers are exposed and affect whether AI has a positive or negative impact on worker health. Based on the findings, we will generate key messages and recommendations that can be used by decision makers to develop strategies relevant to occupations across diverse employment and working conditions with the aim of minimizing AI’s potential workplace harms while amplifying its potential benefits [7].

Inequity is a growing characteristic of many labour market contexts in the Global North [11]. Studies consistently show disparities in the employment and working conditions to which different groups of workers are exposed which contribute to health and social inequities [9, 18, 19]. The introduction of new technologies within the working world have previously disproportionally affected certain groups of workers, with adverse impacts most pronounced among systemically marginalized worker groups (i.e., racialized persons, women and gender minorities, recent immigrants, and persons with disabilities [11, 23, 64, 65]). Through a focus on workers’ social position we will investigate potential worker inequities that may emerge from the adoption of AI systems and identify groups of workers who may be disproportionately affected by the technology. Findings generated through our LSR will enable the development of targeted solutions to address emerging worker inequities as AI systems continue to be implemented. Findings will also inform future priorities for primary research where evidence is critically needed to address worker equity issues.

Our LSR approach has several strengths. The first strength is that the LSR method combines a structured review approach with an a priori commitment to search and identify new evidence as it emerges, making it especially helpful for synthesizing research on a topic in which the evidence is quickly evolving [52, 53]. Including both quantitative and qualitative research captures multiple perspectives on the risks and benefits of AI systems on worker safety, health, and well-being. A final strength of our LSR is adopting an integrated knowledge transfer and exchange approach that includes various workplace and AI stakeholders to provide an applied perspective to study design and interpretation of findings. The integration of diverse stakeholders will increase the relevance and suitability of dissemination materials and uptake of the research evidence. A limitation of the LSR process is that it can be resource intensive and require input from a large multidisciplinary research team. Another limitation is knowing how long to maintain the review in its living mode. While the future of the living review will be assessed annually and be determined by the quality of the emerging evidence and its relevancy to stakeholders, in practice, it is difficult to assess whether new evidence is forthcoming that may change the certainty of the evidence base. Ultimately, the decision will be determined by the core research review team in consultation with key stakeholders.

## Conclusion

Our LSR will capture the changing world of work to which workers are exposed focusing specifically on AI adoption within the workplace and worker safety, health, and well-being. Our LSR will synthesize emerging evidence on the impact of AI systems and produce recommendations that can be used to ensure that the adoption of AI within workplaces may maximize benefits, minimize risks, and safeguard workers [5, 7, 43, 66]. By focusing on workers’ social position, employment and working conditions, in addition to worker health outcomes, our review will generate practical insights that centre workers in a broader discourse on workplace AI advancement and adoption.

## Supplementary Information

Additional file 1: PRISMA-P-SystRev-checklist.

Additional file 2: Search strategy example.

## Data Availability

Not applicable.

## Abbreviation

AI: Artificial intelligence
