# Supplementary material for: Artificial intelligence in the workplace: a living systematic review protocol on worker safety, health, and well-being implications
Source: Syst Rev. 2025 Dec 30;14:255. doi: 10.1186/s13643-025-03000-0 (PMC12754963; doi:10.1186/s13643-025-03000-0)
Supplement: Supplementary file 1 — Additional file 1: PRISMA-P-SystRev-checklist. [file 13643_2025_3000_MOESM1_ESM.docx]

**PRISMA-P 2015 Checklist**

# **This checklist has been adapted for use with protocol submissions to *Systematic Reviews* from Table 3 in Moher D et al**:**** Preferred reporting items for systematic review and meta-analysis protocols (PRISMA-P) 2015 statement. *Systematic Reviews* 2015 ****4****:1

| **Section/topic** | **#** | **Checklist item** | **Information reported** | | **Line number(s)** |
| --- | --- | --- | --- | --- | --- |
|  |  |  | **Yes** | **No** |  |
| **ADMINISTRATIVE INFORMATION** | | | | | |
| **Title** | | | | | |
| Identification | 1a | Identify the report as a protocol of a systematic review | X |  | 1, 45 |
| Update | 1b | If the protocol is for an update of a previous systematic review, identify as such |  |  |  |
| **Registration** | 2 | If registered, provide the name of the registry (e.g., PROSPERO) and registration number in the Abstract | X |  | Prospero, 78 |
| **Authors** | | | | | |
| Contact | 3a | Provide name, institutional affiliation, and e-mail address of all protocol authors; provide physical mailing address of corresponding author | X |  | 5-41 |
| Contributions | 3b | Describe contributions of protocol authors and identify the guarantor of the review | X |  | 475-479 |
| **Amendments** | 4 | If the protocol represents an amendment of a previously completed or published protocol, identify as such and list changes; otherwise, state plan for documenting important protocol amendments |  |  |  |
| **Support** | | | | | |
| Sources | 5a | Indicate sources of financial or other support for the review | X |  | 471-474 |
| Sponsor | 5b | Provide name for the review funder and/or sponsor | X |  | 471-472 |
| Role of sponsor/funder | 5c | Describe roles of funder(s), sponsor(s), and/or institution(s), if any, in developing the protocol |  |  |  |
| **INTRODUCTION** | | | | | |
| **Rationale** | 6 | Describe the rationale for the review in the context of what is already known | X |  | 134-153 |
| **Objectives** | 7 | Provide an explicit statement of the question(s) the review will address with reference to participants, interventions, comparators, and outcomes (PICO) | X |  | 181-186 |
| **METHODS** | | | | | |
| **Eligibility criteria** | 8 | Specify the study characteristics (e.g., PICO, study design, setting, time frame) and report characteristics (e.g., years considered, language, publication status) to be used as criteria for eligibility for the review | X |  | 242-256 |
| **Information sources** | 9 | Describe all intended information sources (e.g., electronic databases, contact with study authors, trial registers, or other grey literature sources) with planned dates of coverage | X |  | 256-258  260-263 |
| **Search strategy** | 10 | Present draft of search strategy to be used for at least one electronic database, including planned limits, such that it could be repeated | X |  | Additional file 2 (and in this document below the checklist) |
| ***STUDY RECORDS*** | | | | | |
| Data management | 11a | Describe the mechanism(s) that will be used to manage records and data throughout the review | X |  | 272-274 |
| Selection process | 11b | State the process that will be used for selecting studies (e.g., two independent reviewers) through each phase of the review (i.e., screening, eligibility, and inclusion in meta-analysis) | X |  | 276-299 |
| Data collection process | 11c | Describe planned method of extracting data from reports (e.g., piloting forms, done independently, in duplicate), any processes for obtaining and confirming data from investigators | X |  | 325-334 |
| **Data items** | 12 | List and define all variables for which data will be sought (e.g., PICO items, funding sources), any pre-planned data assumptions and simplifications | X |  | 242-247 |
| **Outcomes and prioritization** | 13 | List and define all outcomes for which data will be sought, including prioritization of main and additional outcomes, with rationale | X |  | 253-256 |
| **Risk of bias in individual studies** | 14 | Describe anticipated methods for assessing risk of bias of individual studies, including whether this will be done at the outcome or study level, or both; state how this information will be used in data synthesis | X |  | 301-322 |
| ***DATA*** | | | | | |
| **Synthesis** | 15a | Describe criteria under which study data will be quantitatively synthesized | X |  | 355- 357 |
|  | 15b | If data are appropriate for quantitative synthesis, describe planned summary measures, methods of handling data, and methods of combining data from studies, including any planned exploration of consistency (e.g., *I* ^2^, Kendall’s tau) |  |  |  |
|  | 15c | Describe any proposed additional analyses (e.g., sensitivity or subgroup analyses, meta-regression) |  |  |  |
|  | 15d | If quantitative synthesis is not appropriate, describe the type of summary planned | X |  | 330-343 |
| **Meta-bias(es)** | 16 | Specify any planned assessment of meta-bias(es) (e.g., publication bias across studies, selective reporting within studies) |  |  |  |
| **Confidence in cumulative evidence** | 17 | Describe how the strength of the body of evidence will be assessed (e.g., GRADE) | X |  | 301-322 |

**Data-base specific search strategy for Embase**

N=1242

Database: Embase Classic+Embase <1947 to 2024 December 05>

Search Strategy:

--------------------------------------------------------------------------------

1 (Worker? or labo?rer?).ti,ab. (317403)

2 employee?.ti,ab. (80589)

3 employer?.ti,ab. (28936)

4 employment/ (88851)

5 employment.ti,ab. (114685)

6 job?.ti,ab. (108013)

7 occupation*.ti,ab. (265262)

8 work/ (40675)

9 workplace/ (59468)

10 "remote work".ti,ab. (507)

11 "virtual work".ti,ab. (132)

12 "home office?".ti,ab. (1035)

13 "remote office".ti,ab. (11)

14 ("offsite work" or "off-site work").ti,ab. (8)

15 or/1-14 (818062)

16 artificial intelligence/ (91912)

17 "artificial intelligence".ti,ab. (61433)

18 machine learning/ (140625)

19 "machine learning".ti,ab. (134324)

20 "intelligen* algorithm".ti,ab. (1120)

21 "collective intelligence".ti,ab. (348)

22 "prediction machine".ti,ab. (118)

23 "computer heuristic".ti,ab. (0)

24 "expert system".ti,ab. (3331)

25 expert system/ (6000)

26 "fuzzy logic".ti,ab. (3146)

27 fuzzy logic/ (5481)

28 "deep learning".ti,ab. (74448)

29 deep learning/ (68516)

30 "human-machine".ti,ab. (3228)

31 man machine interaction/ (3862)

32 "natural language processing".ti,ab. (10684)

33 natural language processing/ (14564)

34 NLP.ti,ab. (6252)

35 neural network.ti,ab. (91679)

36 artificial neural network/ (62374)

37 smart sensor$.ti,ab. (576)

38 automated reasoning.ti,ab. (107)

39 automated reasoning/ (67)

40 computer vision.ti,ab. (9667)

41 computer vision/ (4961)

42 smart machine$.ti,ab. (46)

43 deep analytics.ti,ab. (7)

44 big data.ti,ab. (16499)

45 decision support system$.ti,ab. (10556)

46 data mining.ti,ab. (17197)

47 data mining/ (21236)

48 multi-agent systems.ti,ab. (818)

49 health informatics.ti,ab. (2705)

50 medical informatics/ (24494)

51 belief state.ti,ab. (32)

52 online agent.ti,ab. (3)

53 learning agent.ti,ab. (134)

54 "strong AI".ti,ab. (25)

55 "weak AI".ti,ab. (8)

56 training data.ti,ab. (14693)

57 predictive analytics.ti,ab. (1177)

58 cognitive automation.ti,ab. (7)

59 intelligent automation.ti,ab. (55)

60 semantic analysis.ti,ab. (921)

61 cognitive computing.ti,ab. (175)

62 AI bias.ti,ab. (37)

63 (AI adj3 (judgment or judgement)).ti,ab. (28)

64 (AI adj3 prediction).ti,ab. (810)

65 automation bias.ti,ab. (77)

66 cloud computing.ti,ab. (2720)

67 data architecture.ti,ab. (107)

68 computational thinking.ti,ab. (57)

69 general adversarial network*.ti,ab. (9)

70 human machine teaming.ti,ab. (21)

71 human AI teaming.ti,ab. (11)

72 intelligent sensing.ti,ab. (174)

73 object recognition.ti,ab. (14368)

74 one shot learning.ti,ab. (130)

75 reinforcement learning.ti,ab. (7696)

76 semi-supervised learning.ti,ab. (1265)

77 explainability.ti,ab. (1709)

78 "generative AI".ti,ab. (701)

79 "large language model*".ti,ab. (3449)

80 or/16-79 (499298)

81 (injur* adj2 preven*).ti,ab. (27785)

82 exp occupational accident/ (29288)

83 occupational accident*.ti,ab. (2290)

84 exp occupational disease/ (176033)

85 occupational disease*.ti,ab. (10022)

86 occupational hazard*.ti,ab. (5250)

87 exp occupational health/ (283055)

88 occupational health.ti,ab. (21453)

89 occupational illness*.ti,ab. (617)

90 occupational injur*.ti,ab. (3581)

91 exp occupational safety/ (15624)

92 occupational safety.ti,ab. (7477)

93 exp work environment/ (42077)

94 work environment.ti,ab. (14857)

95 (workplace* adj2 hazard*).ti,ab. (800)

96 (workplace* adj2 injur*).ti,ab. (1056)

97 workplace health.ti,ab. (2000)

98 (workplace adj2 safety).ti,ab. (1568)

99 working condition*.ti,ab. (18420)

100 work* stress.ti,ab. (4240)

101 job stress.ti,ab. (3115)

102 technostress.ti,ab. (131)

103 (wellbeing or well-being).ti,ab. (203892)

104 psych* injur*.ti,ab. (466)

105 job demand*.ti,ab. (3829)

106 job demands-resources model.ti,ab. (291)

107 effort reward imbalance.ti,ab. (1329)

108 effort-reward imbalance.ti,ab. (1329)

109 job content questionnaire.ti,ab. (665)

110 JCQ.ti,ab. (290)

111 Copenhagen Psychosocial Questionnaire/ (136)

112 ("Copenhagen Psychosocial Questionnaire" or COPSOQ).ti,ab. (345)

113 psychosocial work environment.ti,ab. (856)

114 physical work environment.ti,ab. (228)

115 mental health/ (247681)

116 psychological safety.ti,ab. (1145)

117 safety climate.ti,ab. (1750)

118 job security.ti,ab. (1107)

119 job insecurity.ti,ab. (1178)

120 alienation.ti,ab. (3199)

121 dignity.ti,ab. (11239)

122 exploitation.ti,ab. (30270)

123 wellness.ti,ab. (22142)

124 "quality of life".ti,ab. (657879)

125 quality of life/ (700099)

126 "life satisfaction".ti,ab. (13840)

127 flourish*.ti,ab. (7239)

128 (thriving or thrive).ti,ab. (26282)

129 (purpose adj2 life).ti,ab. (4364)

130 or/81-129 (1781334)

131 15 and 80 and 130 (1852)

132 limit 131 to yr=2019-2024 (1242)
