# Supplementary material for: Artificial intelligence in the workplace: a living systematic review protocol on worker safety, health, and well-being implications
Source: Syst Rev. 2025 Dec 30;14:255. doi: 10.1186/s13643-025-03000-0 (PMC12754963; doi:10.1186/s13643-025-03000-0)
Supplement: Supplementary file 2 — Additional file 2: Search strategy example. [file 13643_2025_3000_MOESM2_ESM.docx]

**Additional file 2: Data-base specific search strategy for Embase as an example of our search approach**

Database: Embase Classic+Embase <1947 to 2024 December 05>

Search Strategy:

--------------------------------------------------------------------------------

1 (Worker? or labo?rer?).ti,ab. (317403)

2 employee?.ti,ab. (80589)

3 employer?.ti,ab. (28936)

4 employment/ (88851)

5 employment.ti,ab. (114685)

6 job?.ti,ab. (108013)

7 occupation*.ti,ab. (265262)

8 work/ (40675)

9 workplace/ (59468)

10 "remote work".ti,ab. (507)

11 "virtual work".ti,ab. (132)

12 "home office?".ti,ab. (1035)

13 "remote office".ti,ab. (11)

14 ("offsite work" or "off-site work").ti,ab. (8)

15 or/1-14 (818062)

16 artificial intelligence/ (91912)

17 "artificial intelligence".ti,ab. (61433)

18 machine learning/ (140625)

19 "machine learning".ti,ab. (134324)

20 "intelligen* algorithm".ti,ab. (1120)

21 "collective intelligence".ti,ab. (348)

22 "prediction machine".ti,ab. (118)

23 "computer heuristic".ti,ab. (0)

24 "expert system".ti,ab. (3331)

25 expert system/ (6000)

26 "fuzzy logic".ti,ab. (3146)

27 fuzzy logic/ (5481)

28 "deep learning".ti,ab. (74448)

29 deep learning/ (68516)

30 "human-machine".ti,ab. (3228)

31 man machine interaction/ (3862)

32 "natural language processing".ti,ab. (10684)

33 natural language processing/ (14564)

34 NLP.ti,ab. (6252)

35 neural network.ti,ab. (91679)

36 artificial neural network/ (62374)

37 smart sensor$.ti,ab. (576)

38 automated reasoning.ti,ab. (107)

39 automated reasoning/ (67)

40 computer vision.ti,ab. (9667)

41 computer vision/ (4961)

42 smart machine$.ti,ab. (46)

43 deep analytics.ti,ab. (7)

44 big data.ti,ab. (16499)

45 decision support system$.ti,ab. (10556)

46 data mining.ti,ab. (17197)

47 data mining/ (21236)

48 multi-agent systems.ti,ab. (818)

49 health informatics.ti,ab. (2705)

50 medical informatics/ (24494)

51 belief state.ti,ab. (32)

52 online agent.ti,ab. (3)

53 learning agent.ti,ab. (134)

54 "strong AI".ti,ab. (25)

55 "weak AI".ti,ab. (8)

56 training data.ti,ab. (14693)

57 predictive analytics.ti,ab. (1177)

58 cognitive automation.ti,ab. (7)

59 intelligent automation.ti,ab. (55)

60 semantic analysis.ti,ab. (921)

61 cognitive computing.ti,ab. (175)

62 AI bias.ti,ab. (37)

63 (AI adj3 (judgment or judgement)).ti,ab. (28)

64 (AI adj3 prediction).ti,ab. (810)

65 automation bias.ti,ab. (77)

66 cloud computing.ti,ab. (2720)

67 data architecture.ti,ab. (107)

68 computational thinking.ti,ab. (57)

69 general adversarial network*.ti,ab. (9)

70 human machine teaming.ti,ab. (21)

71 human AI teaming.ti,ab. (11)

72 intelligent sensing.ti,ab. (174)

73 object recognition.ti,ab. (14368)

74 one shot learning.ti,ab. (130)

75 reinforcement learning.ti,ab. (7696)

76 semi-supervised learning.ti,ab. (1265)

77 explainability.ti,ab. (1709)

78 "generative AI".ti,ab. (701)

79 "large language model*".ti,ab. (3449)

80 or/16-79 (499298)

81 (injur* adj2 preven*).ti,ab. (27785)

82 exp occupational accident/ (29288)

83 occupational accident*.ti,ab. (2290)

84 exp occupational disease/ (176033)

85 occupational disease*.ti,ab. (10022)

86 occupational hazard*.ti,ab. (5250)

87 exp occupational health/ (283055)

88 occupational health.ti,ab. (21453)

89 occupational illness*.ti,ab. (617)

90 occupational injur*.ti,ab. (3581)

91 exp occupational safety/ (15624)

92 occupational safety.ti,ab. (7477)

93 exp work environment/ (42077)

94 work environment.ti,ab. (14857)

95 (workplace* adj2 hazard*).ti,ab. (800)

96 (workplace* adj2 injur*).ti,ab. (1056)

97 workplace health.ti,ab. (2000)

98 (workplace adj2 safety).ti,ab. (1568)

99 working condition*.ti,ab. (18420)

100 work* stress.ti,ab. (4240)

101 job stress.ti,ab. (3115)

102 technostress.ti,ab. (131)

103 (wellbeing or well-being).ti,ab. (203892)

104 psych* injur*.ti,ab. (466)

105 job demand*.ti,ab. (3829)

106 job demands-resources model.ti,ab. (291)

107 effort reward imbalance.ti,ab. (1329)

108 effort-reward imbalance.ti,ab. (1329)

109 job content questionnaire.ti,ab. (665)

110 JCQ.ti,ab. (290)

111 Copenhagen Psychosocial Questionnaire/ (136)

112 ("Copenhagen Psychosocial Questionnaire" or COPSOQ).ti,ab. (345)

113 psychosocial work environment.ti,ab. (856)

114 physical work environment.ti,ab. (228)

115 mental health/ (247681)

116 psychological safety.ti,ab. (1145)

117 safety climate.ti,ab. (1750)

118 job security.ti,ab. (1107)

119 job insecurity.ti,ab. (1178)

120 alienation.ti,ab. (3199)

121 dignity.ti,ab. (11239)

122 exploitation.ti,ab. (30270)

123 wellness.ti,ab. (22142)

124 "quality of life".ti,ab. (657879)

125 quality of life/ (700099)

126 "life satisfaction".ti,ab. (13840)

127 flourish*.ti,ab. (7239)

128 (thriving or thrive).ti,ab. (26282)

129 (purpose adj2 life).ti,ab. (4364)

130 or/81-129 (1781334)

131 15 and 80 and 130 (1852)

132 limit 131 to yr=2019-2024 (1242)
